# Goldberg–Shprintzen syndrome is determined by the absence, or reduced expression levels, of KIFBP

**DOI:** 10.1002/humu.24097

**Published:** 2020-09-16

**Authors:** Katherine C. MacKenzie, Bianca M. de Graaf, Andreas Syrimis, Yuying Zhao, Erwin Brosens, Grazia M. S. Mancini, Rachel Schot, Dicky Halley, Martina Wilke, Arve Vøllo, Frances Flinter, Andrew Green, Sahar Mansour, Jacek Pilch, Zornitza Stark, Eleni Zamba‐Papanicolaou, Violetta Christophidou‐Anastasiadou, Robert M. W. Hofstra, Jan D. H. Jongbloed, Nayia Nicolaou, George A. Tanteles, Alice S. Brooks, Maria M. Alves

**Affiliations:** ^1^ Department of Clinical Genetics Erasmus University Medical Centre Rotterdam The Netherlands; ^2^ Department of Clinical Genetics The Cyprus Institute of Neurology & Genetics and Archbishop Makarios III Medical Centre Nicosia Cyprus; ^3^ Department of Paediatrics Sykehuset Østfold HF Fredrikstad Norway; ^4^ Department of Clinical Genetics Guy's and St Thomas' NHS Foundation Trust London UK; ^5^ Department of Clinical Genetics Children's Hospital Ireland at Crumlin Dublin Ireland; ^6^ South West Thames Regional Genetic Service, St George's Hospital Medical School London UK; ^7^ Department of Child Neurology Medical University of Silesia Katowice Poland; ^8^ Victorian Clinical Genetics Services, Murdoch Children's Research Institute Melbourne Australia; ^9^ Department of Paediatrics University of Melbourne Melbourne Australia; ^10^ Neurology Clinic D, The Cyprus Institute of Neurology & Genetics Nicosia Cyprus; ^11^ Department of Genetics University Medical Centre Groningen Groningen The Netherlands

**Keywords:** GOSHS, HSCR, KIAA1279, KIFBP, missense variants

## Abstract

Goldberg–Shprintzen syndrome (GOSHS) is caused by loss of function variants in the kinesin binding protein gene (*KIFBP*). However, the phenotypic range of this syndrome is wide, indicating that other factors may play a role. To date, 37 patients with GOSHS have been reported. Here, we document nine new patients with variants in *KIFBP*: seven with nonsense variants and two with missense variants. To our knowledge, this is the first time that missense variants have been reported in GOSHS. We functionally investigated the effect of the variants identified, in an attempt to find a genotype–phenotype correlation. We also determined whether common Hirschsprung disease (HSCR)‐associated single nucleotide polymorphisms (SNPs), could explain the presence of HSCR in GOSHS. Our results showed that the missense variants led to reduced expression of KIFBP, while the truncating variants resulted in lack of protein. However, no correlation was found between the severity of GOSHS and the location of the variants. We were also unable to find a correlation between common HSCR‐associated SNPs, and HSCR development in GOSHS. In conclusion, we show that reduced, as well as lack of KIFBP expression can lead to GOSHS, and our results suggest that a threshold expression of KIFBP may modulate phenotypic variability of the disease.

## 1 INTRODUCTION

Goldberg–Shprintzen syndrome (GOSHS; MIM# 609460) is a rare and severe autosomal recessive disorder, characterized by moderate intellectual disability, dysmorphic facial features (arched eyebrows, dense eyelashes, broad nasal bridge, hypertelorism, synophrys, ptosis, large ears, and a prominent long nose), microcephaly (head circumference < ‐2.5 SD), and axonal neuropathy. GOSHS was first described by Goldberg and Shprintzen in 1981, and to date, 37 cases have been reported through clinical diagnosis, with variable severities and additional features, such as iris coloboma, cleft palate/bifid uvula, corneal ulcers, and congenital heart defects (Breslau and Laan, 1989; Brooks, 2005; Brooks et al., 1999; Brooks et al., 2005; Brunoni, Joffe, Farah, & Cunha, 1983; Bruno et al., 2011; Dafsari et al., 2015; Drévillon et al., 2013; Fryer, 1998; Goldberg & Shprintzen, 1981; Halal & Morel, 1990; Hurst, Markiewicz, Kumar, & Brett, 1988; Kumasaka & Clarren, 1988; Silengo et al., 2003; Ohnuma, Imaizumi, Masuno, Nakamura, & Kuroki, 1997; Murphy, Carver, Brooks, Kenny, & Ellis, 2006; Salehpour, Hashemi‐Gorji, Soltani, Ghafouri‐Fard, & Miryounesi, 2017; Tanaka, Ito, Cho, & Mikawa, 1993; Valence et al., 2013; Yomo, Taira, & Kondo, 1991). Homozygosity mapping followed by Sanger sequencing, identified homozygous or compound heterozygous loss of function (LOF) variants in the kinesin binding protein gene (*KIFBP*, previously known as *KIAA1279*), as causative for GOSHS (Brooks et al., 2005; Dafsari et al., 2015; Valence et al., 2013). Twenty‐five out of the 37 reported cases have been shown to have truncating variants in this gene (Brooks, 2005; Brooks et al., 1999; Brooks et al., 2005; Bruno et al., 2011; Dafsari et al., 2015; Drévillon et al., 2013; Goldberg & Shprintzen, 1981; Hurst et al., 1988; Murphy et al., 2006; Salehpour et al., 2017; Valence et al., 2013). KIFBP is expressed throughout the developing embryo at early stages of development and becomes highly expressed in the central and peripheral nervous systems at later developmental stages (Alves et al., 2010). KIFBP is 621 amino acids long and contains two tetratricopeptide repeats. It is involved in the axonal structure and outgrowth, microtubule dynamics, and cargo trafficking, functioning by binding with various microtubule‐associated proteins, such as kinesins and the superior cervical ganglia 10 (SCG10; Alves et al., 2010; Kevenaar et al., 2016; Lyons, Naylor, Mercurio, Dominguez, & Talbot, 2008; Wozniak, Melzer, Dorner, Haring, & Lammers, 2005). However, the location of the binding domains of KIFBP remains unmapped, and its precise function is unknown. In mice, inactivation of *Kifbp* is lethal, leading to central and peripheral nervous system defects and delayed enteric nervous system development (Hirst et al., 2017). Similarly, knocking out the *KIFBP* orthologue in zebrafish led to a disruption of axonal structure and outgrowth, including axonal defects in the enteric nervous system (Lyons et al., 2008).

Hirschsprung disease (HSCR) is reported in ~70% of patients with GOSHS, but is considered to be a variable feature. HSCR is characterized by the absence of enteric ganglia in the distal colon and occurs in multiple defined syndromes (Amiel et al., 2008). The link between GOSHS and HSCR is poorly understood, especially considering the variability of its presence, even between family members sharing the same pathogenic variant. It is suspected that a balance of protective and/or predisposing factors in the (epi)genome influences HSCR development (Brosens et al., 2016; Chatterjee et al., 2016; Emison et al., 2005; Kapoor et al., 2015). Common variants in the Rearranged during transfection gene (*RET*), the Semaphorin 3A gene (*SEMA3A*), and the Neuregulin 1 gene (*NRG1*) are known to be associated with HSCR risk. Previous studies have already investigated the effect of common variants located in intron 1 of *RET* in a series of patients diagnosed with a Mendelian syndrome where HSCR is part of the phenotype (de Pontual et al., 2006, 2007). However, no such study has been performed yet for GOSHS.

Here, we provide an update of all *KIFBP* reported cases and add nine unpublished cases with six new *KIFBP* variants, three of which are missense. This is the first time that missense variants have been reported to play a role in GOSHS. Whether these missense variants also result in LOF is unknown. Therefore, we functionally tested the effect of these variants, on KIFBP expression levels and cellular localization. We also investigated if the truncating variants described, result in loss of protein. Finally, we determined if the occurrence of HSCR in GOSHS can be explained by the presence of common modifier alleles.

## 2 MATERIALS AND METHODS

### 2.1 Patient inclusion

In this study, nine new patients were included (Table 1). These patients were seen routinely in hospitals in the UK, Ireland, Norway, Poland, Australia, The Netherlands, and Cyprus. Eight of the nine patients were ascertained by clinical diagnosis of GOSHS, followed by molecular confirmation. The criteria used for the clinical diagnosis relied mainly on the presence of microcephaly, dysmorphic facial features, and detection of axonal neuropathy. The presence of HSCR was suspected by the inability to pass meconium on the first days of life, followed by an abdominal X‐ray and a contrast enema. Confirmation of the disease was done by histopathological analysis of a rectal suction biopsy.

**Table 1:** All published and unpublished patients with *KIFBP* variants and their clinical features (ENST00000361983.7)

Code	US1	US2	Case1	Case2	BP1	BP2	V‐4	V‐6	V‐9
Sex	M	F	M	M	F	F	M	F	M
***KIFBP*** **variant**	**c.1551‐1552insA**	**c.1551‐1552insA**	**c.250G**>**T**	**c.250G**>**T**	**c.250G**>**T**	**c.250G**>**T**	**c.268C**>**T**	**c.268C**>**T**	**c.268C**>**T**
**Protein**	p. Gln518 Asnf*11	p. Gln518 Asnf*11	p. Glu84*	p. Glu84*	p. Glu84*	p. Glu84*	p. Arg90*	p. Arg90*	p. Arg90*
**HSCR**	+	+	+	+	+	−	+	+	−
Facial dysmorphism	+	+	+	+	+	+	+	+	+
Microcephaly	+	+	+	+	+	+	−	+	+
Brain malformation	+	+	+	+	+	+	+	+	+
Developmental delay	+	+	+	+	+	+	+	+	+
Seizures	+	−	−	−	−	−	−	−	−
Neuropathy	?	?	?	?	?	?	?	?	?
Short stature	+	+	+	+	+	+	?	+	+
Hypotonia	+	+	?	?	?	?	?	?	+
Eye anomalies (coloboma, ptosis, hyperopia; megalocornea)	+	+	+	+	+	+	?	+	+
Cardiac anomalies (ventricular septal defects, aortic valve incompetence)	−	−	−	−	−	−	?	−	−
Skeletal anomalies (oligodontia, scoliosis)	+	+	+	−	−	−	+	+	−
OFC centile	<2nd centile	<2nd centile	<3rd centile	<3rd centile	3rd centile	?	3rd centile	3rd centile	<3rd centile
**Ref**	Goldberg and Shprintzen (1981)	Goldberg and Shprintzen (1981)	Hurst et al. (1988)	Hurst et al. (1988)	Hurst et al. (1988)	Brooks (2005)	Brooks et al. (1999)	Brooks et al. (1999)	Brooks et al. (1999)

**Code**	**VI‐1**	**VI‐3**	**CYP1**	**CYP2**	**UK1**	**UK2**	**AU1**	**IV‐2**	**IV‐1**
**Sex**	**F**	**F**	**M**	**F**	**M**	**M**	**F**	**M**	**M**
***KIFBP* variant**	**c.268C**>**T**	**c.268C**>**T**	**c.718G**>**T**	**c.718G**>**T**	**c.1117‐1118insA**	**c.1117‐1118insA**	**c.1397dupA**	**c.268C**>**T**	**c.599C**>**A**
**Protein**	p. Arg90*	p. Arg90*	p. Glu240*	p. Glu240*	p. Ala373 Asnf*17	p. Ala373 Asnf*17	p. Tyr466*	p. Arg90*	p. Ser200*
**HSCR**	+	+	+	−	+	+	+	+	+
Facial dysmorphism	+	+	+	+	+	+	+	+	+
Microcephaly	+	+	+		+	+	+	+	+
Brain malformation	+	+	+	+	+	+	+	+	+
Developmental delay	+	+	?	?	+	+	+	+	+
Seizures	−	−	−	−	+	−	?	?	?
Neuropathy	?	?	?	?	?	?	?	?	?
Short stature	+	?	?	?	?	?	+	?	?
Hypotonia	+	?	+	+	+	+	?	?	?
Eye anomalies (coloboma, ptosis, hyperopia; megalocornea)	?	?	?	?	−	−	−	+	+
Cardiac anomalies (ventricular septal defects, aortic valve incompetence)	−	?	?	?	−	−	−	−	+
Skeletal anomalies (oligodontia, scoliosis)	+	?	−	+	+	+	−	−	+
OFC centile	3rd centile	?	<3rd centile	?	<3rd centile	<3rd centile	?	?	?
**Ref**	Brooks et al. (1999)	Brooks et al. (2005)	Brooks (2005)	Brooks (2005)	Murphy et al. (2006)	Murphy et al. (2006)	Bruno et al. (2011)	Drévillon et al. (2013)	Drévillon et al. (2013)

**Code**	**IV‐3**	**IV‐1.2**	**V‐2**	**IV.5**	**IV.8**	**UK7**	**IRN1**	**IE1**	**UK3**
**Sex**	**M**	**F**	**F**	**F**	**F**	**F**	**M**	**F**	**M**
***KIFBP* variant**	**c.599C**>**A**	**c.604_605 delAG**	**c.604_605delAG**	**Deletion exon 2 & 3**	**Deletion exon 2 & 3**	**Deletion exon 5 & 6**	**c.976C**>**T**	**c.1694_1695 delAG**	**Deletion exon 6**
**Protein**	p. Ser200*	p. Arg202 Ilefs*2	p. Arg202 Ilefs*2	**p. Asn143fsX1**	**p. Asn143fsX1**	−	p. Gln326*	**p. Glu565 AsnfX15**	−
**HSCR**	+	−	+	−	?	+	+	+	+
Facial dysmorphism	+	+	+	+	−	+	+	+	+
Microcephaly	+	+	+	+	+	+	+	+	+
Brain malformation	+	+	+	+	+	+	+	+	+
Developmental delay	+	+	+	+	+	+	+	+	+
Seizures	?	?	?	?	?	−	+	?	−
Neuropathy	?	?	?	?	?	+	?	?	+
Short stature	?	?	?	?	?	+	+	?	+
Hypotonia	?	?	?	?	?	+	+	+	?
Eye anomalies (coloboma, ptosis, hyperopia; megalocornea)	+	−	−	?	?	+	+	+	+
Cardiac anomalies (ventricular septal defects, aortic valve incompetence)	−	−	+	?	?	+	−	+	−
Skeletal anomalies (oligodontia, scoliosis)	+	−	−	?	?	+	−	−	+
OFC centile	?	?	?	3rd centile	3rd centile	<0.4th centile	normal	?	<0.4th centile
**Ref**	Drévillon et al. (2013)	Drévillon et al. (2013)	Drévillon et al. (2013)	Valence et al. (2013)	Valence et al. (2013)	Dafsari et al. (2015)	Salehpour et al. (2017)	This paper	This paper

**Code**	**UK4**	**PL1**	**PL2**		**NO1**	**NO2**	**CYP3**	**NL1**
**Sex**	**F**	**M**	**M**		**F**	**M**	**F**	**M**
***KIFBP* variant**	**Deletion exon 6**	**c.1516dupA**	**c.1516dupA**		**Deletion exon 5&6**	**Deletion exon 5&6**	**c. 565C**>**T**	**c.68A**>**G; c.1279A**>**G**
**Protein**	−	p. Ile506 Asnfs*3	p. Ile506Asnfs*3		−	−	**p. Pro189Ser**	**p. Glu23Gly; p. Ser427Gly**
**HSCR**	−	+	−		+	−	+	−
Facial dysmorphism	+	+	+		+	+	+	−
Microcephaly	+	+	+		+	+	+	+
Brain malformation	−	−	+		+	+	+	+
Developmental delay	+	+	+		+	+	+	+
Seizures	−	−	−		−	−	−	−
Neuropathy	?	−	−		−	−	+	?
Short stature	+	+	+		+	+	+	+
Hypotonia	?	−	−		+	+	?	?
Eye anomalies (coloboma, ptosis, hyperopia; megalocornea)	−	+	+		+	+	+	?
Cardiac anomalies (ventricular septal defects, aortic valve incompetence)	−	−	−		−	+	−	?
Skeletal anomalies (oligodontia, scoliosis)	+	+	+		−	+	+	?
OFC centile	<0.4th centile	<3rd centile	<3rd centile		<1st centile	<1st centile	<0.4th centile	?
**Ref**	This paper	This paper	This paper		This paper	This paper	This paper	This paper

Abbreviation: HSCR, Hirschsprung disease.

Seven patients were genetically screened for *KIFBP* variants at the University Medical Centre Groningen, Groningen, NL, to confirm diagnosis of GOSHS. The two patients carrying missense variants were screened at the department of Clinical Genetics in the Erasmus Medical Centre, Rotterdam, NL, and the department of Clinical Genetics at the Cyprus Institute of Neurology and Genetics, in Nicosia, Cyprus. Permission to use diagnostic findings for publication was obtained from all parents.

### 2.2 Sequencing

Sanger sequencing of *KIFBP* was performed for eight of the nine patients, as previously described (Brooks et al., 2005). A list of primers is available on request. Patient NL1 was the only one subjected to whole‐exome sequencing, due to lack of phenotypic features characteristic of GOSHS. Sequencing data were analyzed using a filter for neuronal migration abnormalities, leading to the identification of two heterozygous missense variants in *KIFBP*. Both variants were confirmed by Sanger sequence. All new variants described were submitted to ClinVAr (http://www.ncbi.nlm.nih.gov/clinvar/).

All patients and parents were also Sanger sequenced for the presence of selected common HSCR associated polymorphisms in *RET* (Chatterjee et al., 2016), *NRG1*, and *SEMA3A* Kapoor et al., 2015). Primers used are listed in Table S1.

### 2.3 Expression vectors

The pcDNA–HA–hKIFBP vector was described before (Alves et al., 2010). When produced from this vector, the KIFBP protein contains an N‐terminal HA‐tag. The three missense variants identified were generated by site‐directed mutagenesis on pcDNA–HA–hKIFBP, according to the manufacturer's instructions (QuickChange II Site‐directed Mutagenesis Kit, Agilent Technologies). We used the same vector and procedure to introduce the two frameshifts variants, as well as the two deletions identified. Sanger sequencing confirmed the presence of all the variants in KIFBP. No additional variants were inserted. Primers used are listed in Table S1.

### 2.4 Cell culture and transfection

Human embryonic kidney cells (HEK293) were cultured in Dulbecco's modified Eagle's medium high medium containing 4.5 g/L glucose, l‐glutamine and pyruvate (Gibco), supplemented with 10% fetal calf serum (Gibco) and 1% penicillin/streptomycin (Gibco). Cells were incubated at 37°C and 5% CO_2_. For transient transfection, 3 × 10^5^ cells were seeded per well in 6‐well plates. After 24 h, transfection with expression vectors containing either HA‐tagged wild type or mutated KIFBP complementary DNA (cDNA) was performed using GeneJuice® transfection reagent (Millipore), according to the manufacturer's instructions.

### 2.5 RNA isolation and q‐PCR

RNA was isolated from HEK293 cells transfected with KIFBP wild‐type (WT) and mutant constructs, using the RNAeasy Kit (Qiagen), according to the manufacturer's instructions. cDNA preparation and quantitative polymerase chain reaction (q‐PCR) were performed, in triplicate, as previously described (McCann et al., 2019). A list of primers used can be found in Table S1.

### 2.6 Immunofluorescence and confocal microscopy

Following KIFBP overexpression in HEK293, cells were fixed with 4% paraformaldehyde for 15 min, and made permeable with 1% bovine serum albumin and 0.1% Triton X‐100 in phosphate‐buffered saline (PBS). Cells were stained for HA using the HA‐Tag antibody (C29F4, Cell Signaling Technology, USA) at 1:1500 dilution, and the Cy3 AffiniPure donkey antirabbit IgG, 1:200 dilution (Jackson Immunoresearch, UK). Three wells were fixed for each construct. Cells were imaged on a Leica SP5 confocal microscope.

### 2.7 Cell lysates and western blot analysis

Twenty‐four to 48 h after transfection, cells were washed with PBS and lysed as described before (Alves et al., 2010). Protein quantification was determined using the Pierce Bicinchoninic Acid Protein Assay kit (Thermo Fisher Scientific), and 40 μg of cell lysates were stored in loading buffer at −80°C before they were processed further. Sodium dodecyl sulfate‐polyacrylamide gel electrophoresis followed by western blot analysis was performed using an in‐house anti‐HA antibody, and a GAPDH antibody (Millipore), both at 1:5000 dilution. Secondary antibodies used were the IRDye 680RD goat antimouse and the IRDye 800CW goat antimouse (Li‐Cor), at 1:10,000 dilution.

### 2.8 Statistical analysis

All results are presented as the mean ± standard error of the mean (SEM). All data were analyzed using a two‐tailed Student *t* test. *p* < .05 was considered to be statistically significant.

## 3 RESULTS

### 3.1 Three novel homozygous truncating variants in *KIFBP* were identified in five patients with GOSHS

**Figure 1:**
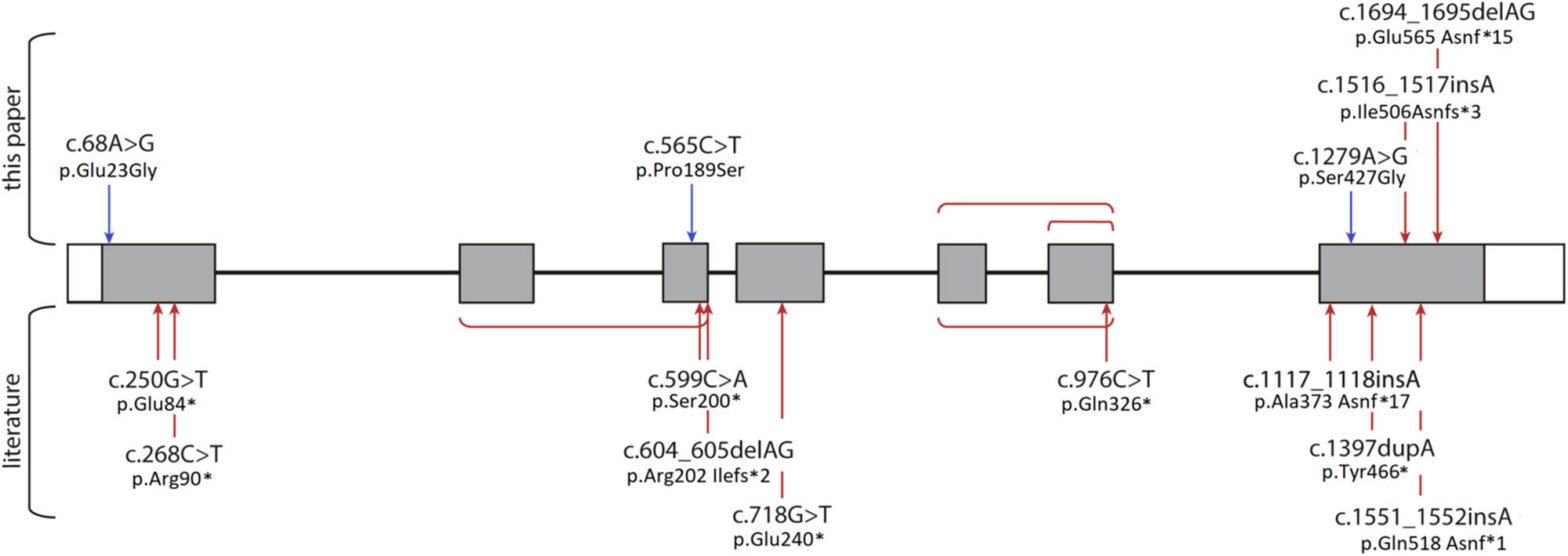
Schematic representation of *KIFBP* highlighting positions of all reported variants. Missense variants are indicated in blue, and nonsense variants in bold red. Square brackets show exon deletions. Transcript number used: ENST00000361983.7.

Seven previously unreported patients with GOSHS, three sibling pairs and an isolated patient, were sequenced for *KIFBP* (Table 1). Large exon deletions, as well as new frameshift variants in *KIFBP*, were identified in these patients (Figure 1 and Table 1). All variants were inherited from healthy parents.

Patient IE1 is a female with a classical GOSHS phenotype and HSCR. She was found to have a homozygous two base‐pair deletion at c.1694_1695 (NM_015634.3:c.1694_1695delAG) of *KIFBP*, causing a premature stop in Exon 7 (accession number: SCV000994837).

Siblings PL1 and PL2, are both males with classical GOSHS facial dysmorphism. PL1 has HSCR, where PL2 does not. A homozygous insertion of an A at position c.1516_1517 of *KIFBP* (NM_015634.3:c.1516dupA) was identified in these patients, leading to the appearance of a premature stop in Exon 7 (Accession number: SCV000994838).

Siblings UK3 and UK4 were found to have a homozygous deletion of Exon 6 of *KIFBP* (NM_015634.3:c.875_990del). Patient UK3 is a male, with classical GOSHS phenotype and HSCR. Patient UK4 is a female and does not have HSCR (accession number: SCV000994839).

Siblings NO1 and NO2 have a deletion of Exons 5 and 6 of *KIFBP* (NM_015634.3:c.790_990del). Both have a classical GOSHS phenotype, but NO1, female, has HSCR where it is absent in her brother, NO2. This deletion is homozygous and has been previously reported (Dafsari et al., 2015).

**Figure 2:**
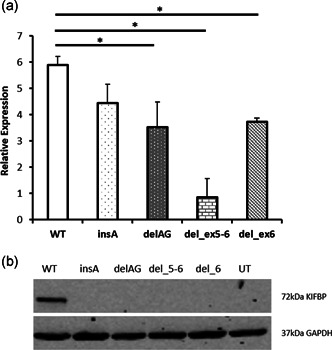
Expression of KIFBP is lost in the presence of the frameshifts and nonsense variants identified. (a) q‐PCR results showing relative normalized expression of *KIFBP* following transfection with wild type (WT) or mutant constructs. All mutant constructs showed a decrease in *KIFBP* expression compared to WT levels. (b) Western blot of KIFBP expression following transfection of either WT or mutant constructs. No KIFBP expression was detected in any of the mutants. q‐PCR, quantitative polymerase chain reaction; UT, untransfected

According to the guidelines established by the American College of Medical Genetics (ACMG), all truncating variants identified in our GOSHS patients are classified as pathogenic (Table S2). These variants are also predicted to result in a total loss of KIFBP expression (Table 2). However, since there is very little information available on the effect of frameshifts and nonsense variants on the stability of KIFBP, we decided to study the effect of these variants, by performing in vitro expression assays. For this, HEK293T cells were transfected with constructs expressing the WT, and mutant KIFBP cDNAs. q‐PCR results showed a decrease on the RNA levels for all mutants analyzed, and no protein expression was detected for any of them. These results show that the truncating variants here described, completely abolish expression of KIFBP (Figure 2a,b).

**Table 2:** *KIFBP* missense variants characteristics and classification

cDNA	Protein	Effect	CADD score	DANN score	GERP++ NR	GERP++ RS	GERP++ RS rank score	MetaLR pred	MetaLR score	MetaSVM pred	MetaSVM score	MutationTaster pred	MutationTaster score	Fathmm‐MKL coding pred	Fathmm‐MKL coding score	gnomAD exomes AC	gnomAD exomes AF	gnomAD genomes AC	gnomAD genomes AF
NM_015634.4:c.68A>G	NP_056449.1:p. Glu23Gly	Missense	28.7	0.998	5.73	5.73	0.896	T	0.1142	T	0.831	D	1	D	0.877	420	0.001707	181	0.005845
NM_015634.4:c. 565C>T	NP_056449.1:p. Pro189Ser	Missense	18.03	0.982	5.79	3.85	0.433	T	0.043	T	1.047	D	0.997	D	0.553	0	0	0	0
NM_015634.4:c.1279A>G	NP_056449.1:p. Ser427Gly	Missense	28.1	0.998	5.62	5.62	0.856	T	0.462	T	0.013	D	1	D	0.993	6	2.44E‐05	2	6.46E‐05

Abbreviations: A, disease‐causing automatic; cDNA, complementary DNA; D, disease causing; T, tolerated.

### 3.2 Three missense *KIFBP* variants were identified in two patients

The first patient (NL1) is a 10‐year‐old male of Moroccan ancestry, born to consanguineous parents. He had a history of microcephaly and presented with short stature. Brain imaging showed pachygyria and he was affected by demyelinating peripheral neuropathy and perceptive deafness. However, he lacked the typical facial features of GOSHS, had no skeletal symptoms, and had no reported gastrointestinal or enteric nervous system abnormalities. As GOSHS was initially not considered a possible diagnosis due to the lack of characteristic features, whole‐exome sequencing was conducted on DNA from blood collected for diagnostic purposes. This resulted in the identification of two heterozygous missense variants in *KIFBP*, one in Exon 1, NM_015634.3:c.68A>G (p. Glu23Gly; Mut1; accession number: SCV000994834), and one in Exon 7, NM_015634.3:c.1279A>G (p. Ser427Gly; Mut2; accession number: SCV000994835; Figure 1; Table 1). The variants were inherited from the parents, and have been previously reported in healthy controls (allele frequency of 0.00223 for Mut1 and 0.0000318 for Mut2 on gnomAD browser). Based on the ACMG guidelines, both variants were predicted to be likely benign (Table S2). However, according to different prediction tools, Polyphen‐2, SIFT, and conservation scores there seems to be evidence to support a deleterious effect on the gene (Table 2). No other likely pathogenic variants were identified in this patient.

The second patient (CYP3), is a 28‐year‐old female of Cypriot ancestry, born to reportedly nonconsanguineous parents. She had a history of microcephaly, mild intellectual disability, and developmental delay. She presented with short stature, typical dysmorphic facial features with bilateral blepharoptosis, and corneal ulcers. She was diagnosed with HSCR at the age of 3 years. In addition, she had scoliosis, lordosis, pes cavus, as well as mild sensory motor neuropathy with both axonal and demyelinating features. Brain magnetic resonance imaging did not reveal a central nervous system (CNS) abnormality. No copy number variations were detected with array‐CGH. Review of the family history revealed that the patient's younger brother was diagnosed with HSCR and died in the neonatal period from sepsis, following surgery for meconium ileus. There was also a report of a maternal relative who apparently died in infancy and had features suggestive of GOSHS. Sequencing of *KIFBP* showed a homozygous missense variant in Exon 3, NM_015634.3:c.565C>T (p. Pro189Ser; Mut3; accession number: SCV000994836; Figure 1; Table 1), which was inherited from the parents. This variant has not been reported before (gnomAD browser), and based on the ACMG guidelines is classified as pathogenic (Table S2). Prediction tools, predict this variant as benign (Table 2). However, its CADD score is 17.95, indicating a potentially pathogenic effect.

### 3.3 KIFBP expression levels were reduced by the missense variants

**Figure 3:**
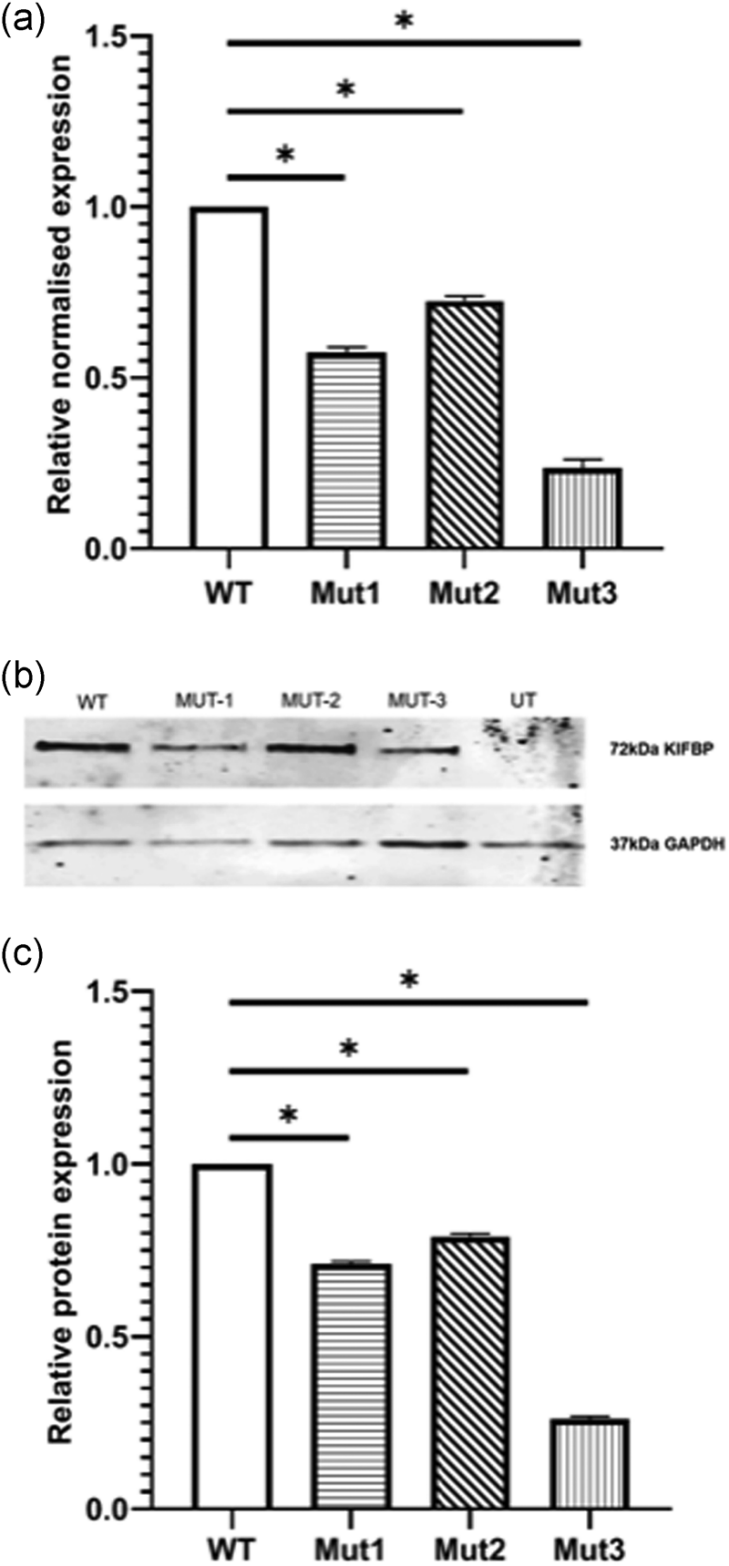
Expression of KIFBP is altered in the presence of the missense variants. (a) q‐PCR results showing relative normalized expression of *KIFBP* following transfection with wild type (WT) or mutant constructs. All mutant constructs show a decrease in *KIFBP* expression compared with WT levels. (b) Western blot of KIFBP expression following transfection of either WT or mutant constructs. Decreased KIFBP expression was detected for all mutants. (c) Quantification of protein expression after normalization for GAPDH. Mut1, A68G, shows ~70% expression, Mut2, A1279G, shows ~80% expression, and Mut3, C565T, shows ~25% expression, when compared with the WT. Error bars show SEM. q‐PCR, quantitative polymerase chain reaction; SEM, standard error of mean; UT, untransfected. *p < .05.

To evaluate the effect of the missense variants, expression levels of KIFBP were determined after the transfection of HEK293T cells with constructs expressing the WT and mutant KIFBP cDNA. q‐PCR results showed significantly decreased RNA levels for all mutants when compared with the wild‐type (Figure 3a). All mutants also showed a significantly decreased protein expression of KIFBP, when compared with WT. But, as expected based on the RNA levels, Mut3 showed the lowest expression (Figure 3b,c).

### 3.4 Cellular localization of KIFBP is unaffected by the missense variants

**Figure 4:**
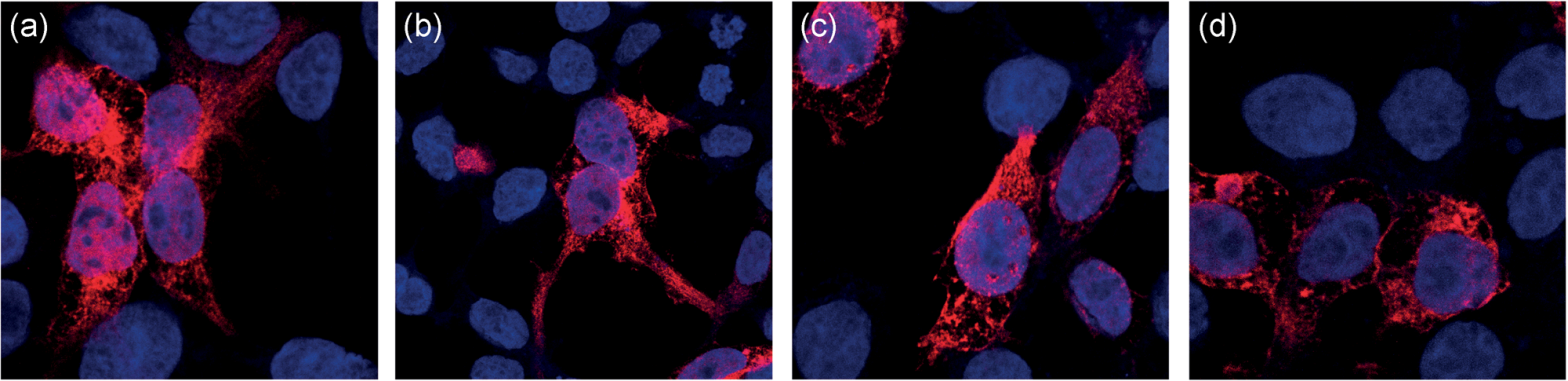
Confocal images of KIFBP localization after transfection of HEK293 cells with wild‐type (WT) and mutant constructs, show no difference between WT and missense variants. (a) WT. (b) Mut1–A68G. (c) Mut2–A1279G. (d) Mut3–C565T

Tagged WT and mutant KIFBP constructs were overexpressed in HEK293 cells, to determine any effect of the variants in the organization or localization of KIFBP within the cell. The WT protein is seen to have high cytoplasmic expression, as previously described (Alves et al., 2010). For the mutant proteins, no effect on KIFBP localization was observed (Figure 4).

### 3.5 Previously associated common SNPs in *RET*, *NRG1*, and *SEMA3A* do not affect HSCR development in GOSHS

It is known that phenotypic variability exists in patients with GOSHS and that HSCR is a variable feature, even within families with the same *KIFBP* truncating variant (Table 1). Here, we investigated whether the presence of previously common SNPs associated with HSCR, would be the determinant factor for the presence of this disorder in GOSHS. The SNPs we decided to investigate are located in intron 1 of *RET*, *SEMA3A*, and *NRG1*. Although, all these SNPS have been described to increase the risk for HSCR (de Pontual et al., 2006, 2007), we were unable to find a significant correlation between them and the occurrence of HSCR in this subset of GOSHS patients and unaffected family members (*t* test, *p* = .526; Tables 3 and 4).

**Table 3:** Influence of common SNPs on the presence of HSCR in GOSHS

		RET	RET	RET	RET	RET	NRG1	NRG1	SEMA3A
#	HSCR	rs2506030	rs7069590	rs2505998	rs2435357	rs9282834	rs1176600	rs8022714	rs7005606
1	No	G/G	T/C	A/G	A/G	G/G	A/A	C/C	G/T
2	No	G/G	T/C	A/G	A/G	G/G	A/A	C/C	T/T
3	No	G/G	T/C	A/G	A/G	G/G	A/A	C/C	T/T
4	No	G/A	C/C	G/G	G/G	G/G	A/C	C/C	G/T
5	No	G/G	T/T	A/G	A/G	G/G	A/A	C/C	G/T
6	No	G/A	T/C	G/G	G/G	G/G	A/A	C/C	T/T
7	No	G/A	T/T	A/A	A/A	G/G	A/A	C/C	T/T
8	No	A/A	T/C	G/G	G/G	G/G	A/C	C/C	G/T
9	No	A/A	T/T	G/G	G/G	G/G	A/A	C/C	G/T
10	No	G/A	T/T	G/G	G/G	G/G	A/A	C/C	G/T
11	No	G/A	T/T	G/G	G/G	G/G	A/A	C/C	T/T
12	No	G/A	T/T	G/G	G/G	G/G	A/A	C/C	T/T
13	No	A/A	T/T	A/A	A/A	G/G	A/A	C/C	T/T
14	No	A/A	T/C	A/G	A/G	G/G	C/C	C/C	T/T
15	Yes	G/G	T/C	A/G	A/G	G/G	A/A	C/C	G/T
16	Yes	G/G	T/C	A/G	A/G	G/G	A/A	C/C	G/T
17	Yes	G/G	T/C	A/G	A/G	G/G	A/A	C/C	T/T
18	Yes	G/G	T/C	A/G	A/G	G/G	A/A	C/C	G/T
19	Yes	G/A	T/C	A/G	A/G	G/G	A/A	C/C	G/G
20	Yes	G/A	T/C	G/G	G/G	G/G	A/C	C/C	G/T
21	Yes	G/A	T/C	A/G	A/G	G/G	A/A	C/C	G/T
22	Yes	G/A	T/C	A/G	A/G	G/G	A/A	C/C	T/T
23	Yes	A/A	T/T	G/G	G/G	G/G	A/A	C/C	T/T
24	Yes	G/A	T/C	G/G	G/G	G/G	A/A	C/C	T/T
25	Yes	G/A	T/T	G/G	G/G	G/G	A/A	C/C	G/G
26	Yes	A/G	T/C	A/G	A/G	G/G	‐	‐	‐

*Note*: Samples are anonymized. Samples 1–14 are unaffected parents. Samples 15–26 are patients with GOSHS with HSCR. Sample 26 was excluded from statistical analysis as data was not available for all SNPs.

Abbreviations: GOSHS, Goldberg–Shprintzen syndrome; HSCR, Hirschsprung disease; SNPs, single nucleotide polymorphisms.

**Table 4:** Statistical analysis shows no correlation between the presence/absence of common SNPs in *RET*, *NRG1*, and *SEMA3A* and HSCR development in patients with GOSHS

Groups	Count	Sum	Average	Variance	ANOVA	SS	*p*‐value
No HSCR	14	63.17114	4.512224	0.179667	Between groups	0.090464	.526023
HSCR	11	50.9675	4.633409	0.268306	Within groups	5.018733	
					Total	5.109196	

Abbreviations: ANOVA, analysis of variance; GOSHS, Goldberg–Shprintzen syndrome; HSCR, Hirschsprung disease; SNPs, single nucleotide polymorphisms.

## 4 DISCUSSION

In this manuscript, we report nine new patients with variants in *KIFBP*. A common feature in all these patients is the presence of intellectual disability and developmental delays. However, the phenotypic spectrum is broad, with distinct facial morphology, microcephaly, and other CNS malformations. Interestingly, this wide phenotypic range is even found in siblings carrying the same variant. As can be seen in Table 1, the incidence of HSCR in GOSHS is ∼70% (24/34). Seven out of 10 patients without HSCR, have a family member carrying the same *KIFBP* variant, that does have this disease, suggesting the presence of modifying factors, or the absence of protective factors in these patients than can tilt the balance in favor of HSCR. Here, we hypothesized that selected common HSCR modifier variants in *RET* (Chatterjee et al., 2016 *)*, *NRG1*, and *SEMA3A* (Kapoor et al., 2015) may work as these modifying factors, as it has been shown for other syndromes (Chatterjee et al., 2016; De Pontual et al., 2007; Tang et al., 2016). However, our results did not show any correlation between the incidence of HSCR and the presence of these common polymorphisms in the cohort analyzed (*p* = .526, Table 3). As HSCR is a complex genetic disease, multiple factors are known to play a role in its development in addition to genetic risk factors, such as epigenetic changes (Tang et al., 2013), protective pathways (Griseri et al., 2007), threshold numbers of cells (Barlow, Wallace, Thapar, & Burns, 2008), or stochastic chance (Cheeseman, Zhang, Binder, Newgreen, & Landman, 2014). Moreover, since HSCR is such a common feature in GOSHS, one might argue that its occurrence is coupled to the expression levels of KIFBP, or to changes in the signaling network regulated by this protein. Further research is therefore required, to investigate which of these hypotheses can explain the variability of such features.

LOF variants in *KIFBP* are known to cause GOSHS. However, there seems to be no correlation between the location of the variant, and the severity of syndromic characteristics (Figure 1), as they all seem to result in total loss of protein (Brooks et al., 2005; Drévillon et al., 2013). In seven of the nine patients reported here, nonsense variants or frameshifts were identified, resulting in loss of expression of *KIFBP* (Figure 2). Unfortunately, we were not able to assess if this was the result of nonsense mediated decay, as we were not able to obtain patient fibroblasts. In the remaining two patients, missense variants were identified. This finding was quite interesting, as no missense variants have been previously reported in GOSHS. Patient CYP3 carries a homozygous variant in *KIFBP* that leads to the amino acid substitution of proline by a serine. Patient NL1 is compound heterozygous, and has two different missense mutations in each of his alleles. One leads to the substitution of glutamate by glycine, while the other changes a serine into glycine. Based on the chemical properties of the different amino acids, the most dramatic change is expected to be the one found in patient CYP3. This is because proline is a nonpolar amino acid that is normally present buried within the protein core due to its hydrophobic nature, while serine is a polar, hydrophilic aminoacid, able to form hydrogen bonds. For patient NL1, although the amino acid changes identified lead to the substitution of relatively big amino acids, glutamate and serine, by glycine, the smallest amino acid due to its minimal side chain, they can basically fit into hydrophilic or hydrophobic environments with relatively minor consequences. Prediction tools and conservation scores are shown in Table 2. We showed here that these missense variants lead to decreased KIF1BP expression, and thus, considered them to be LOF as well. However, the characteristics and diagnosis of these two patients differ tremendously. While patient CYP3 has the hallmark features of GOSHS, including HSCR (Table 1), patient NL1 does not show any of the clinically defined features of this syndrome. In fact, the variants in *KIBBP* were only identified in this patient after exome sequence, and we cannot exclude that they are just polymorphisms due to the fact that they have both been found in the general population. However, our functional results suggest otherwise, as a reduction of KIFBP expression was detected for the three variants tested (Figure 3a–c), suggesting that a threshold expression of this protein may be required for regulation of developmental functions. While patient NL1 shows a mild reduction of KIFBP expression that seems to be, to some extent, tolerable, in patient CYP3 this threshold was not reached, leading to the typical GOSHS phenotype. Therefore, we conclude that missense variants in *KIFBP* can be as damaging as truncating variants, depending on its effect on protein expression levels.

It has been previously noted that the diagnosis of GOSHS should rely on molecular and genetic findings in place of phenotypic recognition only, due to its similarity with other syndromes (Salehpour et al., 2017). Based on our genetic findings, patient NL1 would be considered to have GOSHS due to the fact that no likely pathogenic variant has been identified in any other gene. However, this patient has no hallmark features of GOSHS. Since locus heterogeneity is lacking in this syndrome, and all patients with the typical features have *KIFBP* variants, we believe that the accurate classification of GOSHS based on phenotype by a clinical geneticist may be more useful for the family to appropriately meet the needs of the patient, as well as for advising clinical treatment.

## CONFLICT OF INTERESTS

All the authors declare that there are no conflict of interests.

## Supporting information

Supporting information

## Data Availability

Cell lines and expressing constructs are available upon request.
