# Supplementary material for: Goldberg–Shprintzen syndrome is determined by the absence, or reduced expression levels, of KIFBP
Source: Hum Mutat. 2020 Sep 16;41(11):1906–17. doi: 10.1002/humu.24097 (PMC7693350; doi:10.1002/humu.24097)
Supplement: Supplementary file 1 — Supporting information [file HUMU-41-1906-s001.docx]

**Supplementary table 1**: List of primers used in this study.

| Name | Primer sequence (5’-3’) |
| --- | --- |
| rs2506030_1FW | GGAGGCGCATCCCTAGCC |
| rs2506030_1RV | CAGCTACTCTGGGGCCTTGC |
| rs7069590_3FW | CAATGGTCCCCTGCACACC |
| rs7069590_3RV | GCACCCCTGGCAGTGACC |
| rs2505998_1FW | TGGCTACCTAGGCTACACACTCAGG |
| rs2505998_1RV | CCCCCAGACCTTTTTCCAAGG |
| rs2435357_1FW | CAGCTGCTGCAGAGTTAATCACC |
| rs2435357_1RV | AGAGGCACCAGGGTCAAAGC |
| rs9282834_1FW | GTCCATGCCTTCCCCACTCC |
| rs9282834_1RV | GGGAAAGTCTGTGTGGAAAACTGC |
| rs11766001_1FW | CAATCAAAATGCAAGACACCATTAGC |
| rs11766001_1RV | TGAAAGATGATGGTGTGGATGAGC |
| rs80227144_1FW | GGGCAGATGGATATGTAGGC |
| rs80227144_1RV | TTGAATAAAATGTCTTATTGTTTTCC |
| rs7005606_2FW | TCTGCACCATAATTACAGCAATGG |
| rs7005606_2RV | TGGAGGGTACCACTTCTAGTTTTGC |
| KBP(A68G)F | TCGCGGGTGGGACTGCATAAAAATCC |
| KBP(A68G)R | CAGAGCGAGCGCCGCCTG |
| KBP(A1279G)F | CCAAGACCACGGTGCTCTGTT |
| KBP(A1279G)R | ACAACTTCAATATGGTCAGTGAC |
| KBP(dupA)F | AAAATAATCTTAATAAGTCAGCAC |
| KBP(dupA)R | TTTTTTTACAATGTGTGAATCAG |
| KBP(delAG)F | CTGGAAAATTTGGCAACATC |
| KBP(delAG)R | CTTTCTTGGGATCTGCAG |
| KBP(del_ex5-6)F | GACAACATAGGAGAGCTTG |
| KBP(del_ex5-6)R | CTTATTGATGTAAAACTGTGAC |
| KBP(del_ex6)F | GACAACATAGGAGAGCTTG |
| KBP(del_ex6)R | TGTCTTCTGTGGCTGAGATC |
| KBP(qPCR)F | TGCACAGGCTTACCTAGAGTCAT |
| KBP(qPCR)R | TCTTCAGGAAGAAAACGCTCA |

**Supplementary table 2**: Classification of the *KIFBP* variants identified according the ACMG guidelines (PVS1: very strong level of pathogenicity; PS: strong level of pathogenicity; PP: supporting pathogenic; BP: supporting benign impact; BS: strong benign; PM: medium level of pathogenicity).

| Type of variant | Classification | Criteria used |
| --- | --- | --- |
| Frameshift/ | Pathogenic | PVS1: null variant in a gene where LOF is a |
| Nonsense |  | known mechanism of disease. |
|  |  | PS3: well established in vitro or in vivo functional studies supportive of a damaging effect. |
|  |  | PM3: for recessive disorders, detected in trans with a pathogenic variant. |
|  |  | PP3: multiple lines of computational evidence support a deleterious effect. |
|  |  | PP4: patient’s phenotype or family history is highly specific for a disease with a single genetic etiology. |
| Compound heterozygous | Likely benign | BP1: missense variant in a gene for which |
| missense |  | primarily truncating variants are known to |
|  |  | cause disease. |
|  |  | BS2: observed in healthy adult individual with full penetrance expected at an early age. |
|  |  | PP3: multiple lines of computational evidence support a deleterious effect. |
| Homozygous missense | Pathogenic | PS3: well established *in vitro* or *in vivo* |
|  |  | functional studies supportive of a damaging |
|  |  | effect. |
|  |  | PM2; absent from controls in Exome |
|  |  | Sequencing project, 1000 Genomes Project, |
|  |  | or Exome Aggregation Consortium. |
|  |  | PM3: for recessive disorders, detected in trans with a pathogenic variant. |
|  |  | PP3: multiple lines of computational evidence support a deleterious effect. |
|  |  | PP4: patient’s phenotype or family history is highly specific for a disease with a single genetic etiology. |
|  |  | BP1: missense variant in a gene for which primarily truncating variants are known to cause disease. |
